# Mapping burnt areas using very high-resolution imagery and deep learning algorithms - a case study in Bandipur, India

**DOI:** 10.1371/journal.pone.0327125

**Published:** 2025-07-16

**Authors:** Sai Balakavi, Vineet Vadrevu, Kristofer Lasko

**Affiliations:** 1 Universities Space Research Association (USRA) Science and Technology Institute, Huntsville, Alabama, United States of America; 2 The University of Alabama in Huntsville, Huntsville, Alabama, United States of America; 3 James Clemens High School, Madison, Alabama, United States of America; 4 Geospatial Research Laboratory, Engineer Research and Development Center, US Army Corps of Engineers, Alexandria, Virginia, United States of America; Shandong Agricultural University, CHINA

## Abstract

Burnt area (BA) mapping is crucial for assessing wildfire impact, guiding restoration efforts, and improving fire management strategies. Accurate BA data helps estimate carbon emissions, biodiversity loss, and land surface properties post-fire changes. In this study, we designed and evaluated two deep learning-based architectures, a Custom UNET and a novel UNET-Gated Recurrent Unit (GRU), for burnt area classification using PlanetScope data over Bandipur, India. Both models demonstrated high accuracy in classifying burnt and unburnt areas. Performance metrics, including Precision, Recall, F1-Score, Accuracy, Mean Intersection over Union (IoU), and Dice Coefficient, revealed that the UNET-GRU hybrid consistently outperformed the Custom UNET, particularly in Recall and spatial overlap metrics. The Receiver Operating Characteristic (ROC) curve indicated excellent classification performance for both models, with the UNET-GRU achieving a higher AUC (0.98) compared to the Custom UNET (0.96). These findings highlight the UNET-GRU’s enhanced capacity to handle finer distinctions and capture spatial and contextual features, making it a robust choice for burnt area classification in the study area. While both models avoided overfitting and maintained generalizability, integrating GRU into the UNET architecture proved particularly effective for precise classification and spatial accuracy. Our results highlight the potential of the novel UNET-GRU for burnt area mapping using very high-resolution data.

## Introduction

Vegetation fires play a critical role in many ecosystems while influencing land cover patterns, including in South and Southeast Asian countries [1–3]. In Asia, particularly in India, they are a primary environmental concern due to diverse ecosystems and climatic conditions, which make these regions highly susceptible to fire outbreaks. The fires, often driven by dry vegetation and human activities, affect forested areas, agricultural landscapes, and grasslands, causing severe ecological, economic, and health issues [4,5]. On the one hand, fires can sometimes be beneficial by promoting seed germination, recycling nutrients, and maintaining biodiversity. In contrast, uncontrolled or intense fires can have devastating ecological, social, and economic consequences. They threaten wildlife and habitats, contribute to air pollution and carbon emissions, and cause soil degradation [6,7]. These fires can also disrupt ecosystem services and exacerbate biodiversity decline [8]. Additionally, they emit significant amounts of greenhouse gases and particulate matter, contributing to deteriorating air quality and global climate change [9].

Understanding, monitoring, and managing vegetation fires are essential for mitigating their adverse impacts and building resilience in fire-prone landscapes [10]. Advanced technologies, including satellite remote sensing and predictive modeling, are increasingly employed to enhance fire detection, risk assessment, and response strategies [11]. Satellite data are indispensable for mapping and monitoring fires and burnt areas, offering comprehensive, timely, and reliable evaluations over large, often inaccessible regions. Satellite imagery also offers varying spatial resolutions, from coarse resolution for large-scale monitoring to high resolution proper for detailed mapping of burnt areas and assessing impacts. For example, multi-spectral satellites such as MODIS and Landsat help capture active fires and burnt areas across various wavelengths [12,13]. These sensors can differentiate between burnt and unburnt areas, detect vegetation stress caused by fires, and assess post-fire regrowth, providing a deeper understanding of fires’ ecological and environmental consequences. High-resolution datasets, such as those from PlanetScope and Maxar, are particularly valuable for delineating burnt areas with precision, quantifying the extent of damage, and monitoring recovery processes over time [14].

Integrating satellite data with advanced analytical techniques, including machine learning and deep learning algorithms, has significantly improved the accuracy and efficiency of fire detection, burnt area mapping, and post-fire impact assessments [15,16]. Recent algorithms such as Decision Level Super Resolution (DLSR)-FireCNet demonstrate the efficacy of integrating medium resolution bi-temporal pre-and post-fire imagery, for burnt area mapping, with high overall accuracy including better preservation of burnt area edges and reduction of missed detections and false alarms [17]. These methods utilize vast amounts of satellite data to automate processes, identify patterns, and generate actionable insights at an unprecedented scale.

In addition to deep learning methods, traditional remote sensing techniques have been widely used for burned area mapping. For instance, burned area algorithms use spectral indices, such as the Normalized Burn Ratio (NBR) and its derivatives, to quantify fire severity and spatial extent [18]. Spectral indices such as the Normalized Burn Ratio (NBR), differenced NBR (dNBR), and relative dNBR (RdNBR) are commonly employed to assess fire severity and extent [19,20]. Other indices like Burn Area Index [21], Normalized Difference Vegetation Index (NDVI) [22], and Mid Infrared Burn Index (MIRBI) [23] have also proven effective, particularly when paired with classifiers such as Random Forest and SVM [24,25].

At large spatial scales, the MODIS burned area product**s** developed by [26,27] have become a global standard. Their algorithms integrate reflectance changes and active fire data for consistent, daily burned area mapping. In addition, change detection methods and time series analyses further support fire monitoring in dynamic landscapes [28]. While these traditional techniques are computationally efficient and interpretable, they may be limited by sensor resolution, atmospheric conditions, or spectral confusion in heterogeneous landscapes. Integrating these methods with modern machine learning and deep learning frameworks including multi-satellite remote sensing data presents opportunities to enhance accuracy and scalability in burnt area mapping.

For example, recent advances in deep learning [29,30], such as the Quadratic Morphological Deep Neural Network [31], have demonstrated the effectiveness of fusing radar and optical satellite data for burned area mapping, emphasizing the value of multimodal integration in improving segmentation accuracy. The Quadratic Morphological Deep Neural Network applies layered neural network architectures to extract and analyze complex spatial and spectral patterns from remote sensing imagery. In the context of environmental monitoring, deep learning algorithms have exhibited strong performance across applications including land use and land cover classification, vegetation health assessment, and wildfire-related analysis [32–35]. Specifically, for accurate identification of burnt areas, deep learning models such as Convolutional Neural Networks (CNNs) are particularly effective in capturing spatial textures and patterns present in multispectral and high-resolution satellite data [36].

However, using deep learning models for burnt area mapping requires rigorous testing and validation to ensure their reliability and accuracy. Testing these algorithms under diverse environmental conditions, across various vegetation types, and using different satellite datasets is essential. Without thorough testing, deep learning models may produce inaccurate results or fail to generalize to new areas, limiting their practical utility. In this study, we address key challenges in burnt area mapping by developing two novel deep learning architectures optimized for processing very high-resolution satellite imagery. The deep learning models are specifically designed to leverage the rich spectral and spatial information in PlanetScope (3 m) imagery, enabling fine-scale delineation of burned areas in heterogeneous landscapes. We apply and rigorously evaluate these architectures over the Bandipur forest region in southern India, a fire-prone landscape characterized by complex topography and burnt area characteristics. We addressed two primary objectives: (1) assess and compare the performance of the proposed deep learning models in segmenting burned areas with high accuracy, and (2) explore their ability to generalize across spatial contexts and detect error patterns. In addition to standard evaluation metrics such as precision, recall, F1-score, Intersection over Union (IoU), etc., we conduct a detailed spatial analysis of misclassification patterns to understand where and why each model succeeds or fails. We also investigate how specific architectural enhancements, such as multi-scale feature extraction, attention mechanisms, and skip connections, improve the models’ capacity to capture the subtle spectral-spatial variations characteristic of post-fire landscapes. The results reveal both the strengths and limitations of deep learning approaches for burnt area delineation in the study area. Our findings highlight the value of high-resolution data and advanced architectures for operational fire monitoring and provide a foundation for future research to improve burnt area mapping across diverse ecosystems.

### Study area

**Fig 1:**
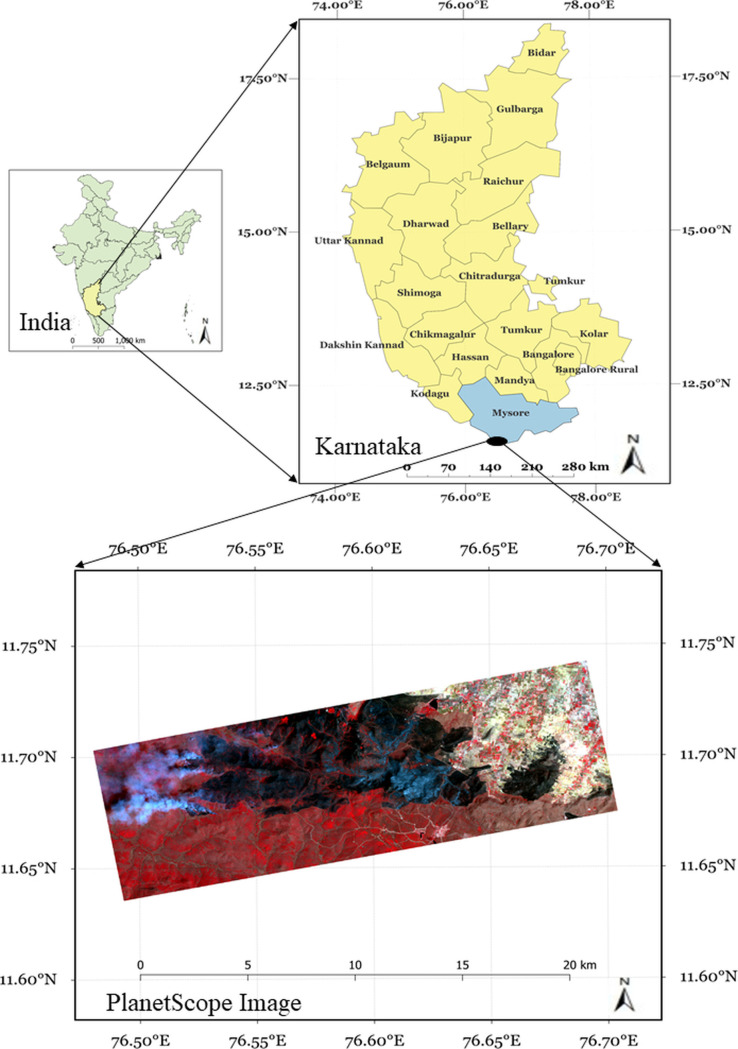
Study area. The green map on the left shows India, with Karnataka State highlighted in yellow. On the right, Karnataka’s districts are shown, with the Mysore district in blue and the Bandipur forest study area highlighted in black. The bottom image is a 3m PlanetScope satellite image of Bandipur National Park, acquired on February 25, 2019.

Bandipur forest, located in the southern Indian state of Karnataka, stands as one of the region’s most prominent national parks and tiger reserves (Fig 1). Bandipur is an integral part of the Nilgiri Biosphere Reserve, globally recognized for its exceptional biodiversity. The park is home to a wide range of flora and fauna, including iconic species such as tigers, elephants, leopards, and numerous bird and plant species. Its diverse ecosystems are vital for maintaining ecological balance in the region, serving as a critical wildlife corridor supporting many species’ movement and survival. Bandipur faces increasing threats from forest fires, especially during the dry season. These fires, which can result from human activities and natural causes, seriously challenge the park’s delicate ecosystems. Addressing this issue requires advanced monitoring and management strategies to mitigate the impact of fires on the forest’s biodiversity. High-resolution satellite data combined with the latest and robust algorithms can provide valuable information for understanding the extent and effects of forest fires in the park, contributing to more effective conservation efforts.

### Datasets

In our study, we utilized high-resolution datasets from the PlanetScope satellite constellation to map burnt areas. Operated by Planet Labs, PlanetScope is renowned for its ability to provide daily global imagery with exceptional spatial resolution ranging between 3 and 5 meters. This capability stems from a network of small, agile satellites ensuring frequent and consistent surface coverage. Such high temporal and spatial resolution makes PlanetScope data invaluable for various applications, including land use/land cover mapping, agricultural monitoring, disaster response, and, notably, burnt area mapping.

For this study, we focused on PlanetScope (3m) surface reflectance imagery with four bands (nir, red, green, and blue) over Bandipur, India, captured on February 25, 2019. Using this dataset, we employed state-of-the-art deep learning algorithms to identify and map burnt areas within the region. For this study, no permits or field work were necessary due to the availability of the very high-resolution 3m PlanetScope data. We focused on evaluating deep learning algorithms for the burnt area delineation, and no deviations from the original protocol were made till the end of the study. Combining PlanetScope’s high-quality imagery and advanced analytical techniques offers critical insights into fire-affected landscapes, supporting effective conservation and restoration strategies.

## Methods

Deep learning is a specialized branch of machine learning that employs layered neural network architectures to uncover and learn intricate patterns from data [37]. Training the neural network models involves techniques like back propagation and gradient descent, which iteratively adjust network weights to optimize performance on large datasets. The multi-layered design enables each layer to build upon the knowledge of the previous one. Deep learning has become indispensable in computer vision and natural language processing, thanks to its ability to extract complex and meaningful features [38]. Popular architectures include Autoencoders [39,40], Convolutional Neural Networks (CNN) [41,42], Recurrent Neural Networks (RNN) [43,44], Long Short-Term Memory (LSTM) networks [45,46], Gated Recurrent Unit (GRU) [47,48], Transfer Learning [49,50], and Transformer architectures [51,52].

The specific training configurations and hyper-parameters used for our models are summarized in Table 1 below.

**Table 1:** Hyperparameter configurations and callbacks used during the training process.

Hyperparameter	Values
Learning Rate	1e^-4^
Number of epochs	50,100
Batch Size	4
Dropout rate	0.2
Optimizer	Adam
Kernel Size	3
Input Size	(256,256,3)
Loss Function	Categorical Cross Entropy
Metrics	Accuracy, IoU-score, Precision, Recall, Dice- Coefficient, ROC-AUC curve
Activation Functions	ReLU, Softmax
CallBacks	Model Checkpoint, Early Stopping

### Traditional UNET architecture overview

**Fig 2:**
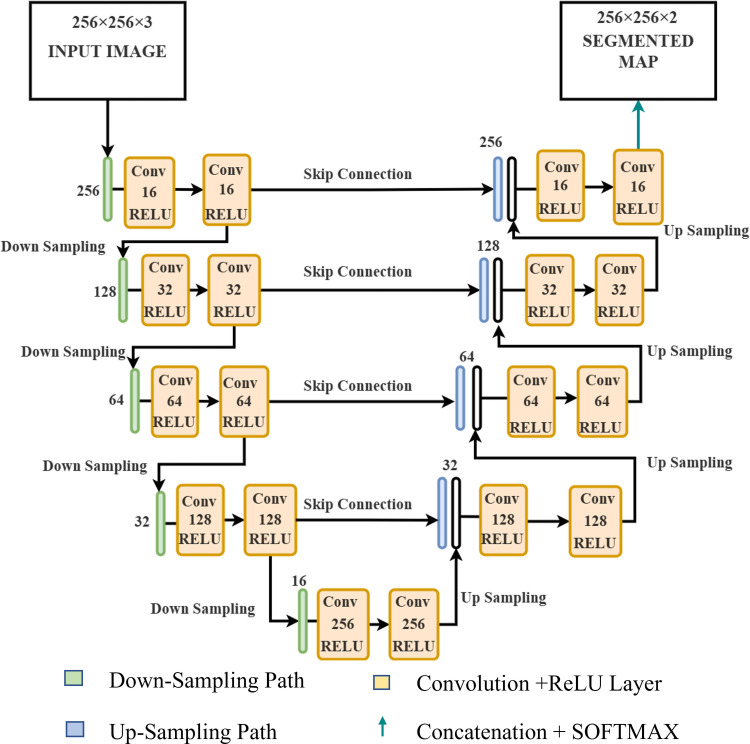
Custom UNET architecture used in the training process for the encoder, decoder, filters, and activation functions.

UNET is a novel approach to semantic segmentation tasks based on Fully Convolutional Neural Networks (FCNN) [53,54], which is used as a base model for this study. We implemented an enhanced version of the Traditional UNET to improve its functionality and segment burnt and unburnt areas in the PlanetScope imagery, as shown in (Fig 2).

### Custom UNET architecture

The architecture of UNET has encoders and decoders. The encoder is a contracting path where image downsampling and feature extraction occur. The input layer is processed with images of size 256 × 256 with three channels. The encoder has four convolutional blocks, each with two 3 × 3 convolutional layers. This is followed by ReLU activation and He-normal weight initialization for adequate gradient flow. This is followed by a max-pooling operation after each convolution block with a size of 2 × 2 to reduce the spatial dimensions by a factor of 2.

In our architecture, the number of filters is doubled after each step of the encoder, starting from 16 and reaching 256 at the bottleneck. Increasing the number of filters after each step ensures that the model captures critical hierarchical features. A dropout rate of 20% is applied after each convolutional block to prevent overfitting. The bottleneck has two convolutional layers with 256 filters, followed by a 20% dropout, as shown in (Fig 2), ensuring the model can capture critical features.

The decoder has the same structure as the encoder, forming the expansive path where upsampling occurs. Each block in the decoder begins with a 2 × 2 transposed convolutional layer, which upsamples the image with a stride of 2 to restore the spatial dimensions. After each upsampling operation, the output from the corresponding encoder block is concatenated with the output from the decoder block via skip connections, as shown in (Fig 2). These skip connections help preserve the spatial information from the encoder, helping to overcome the problem of vanishing/exploding gradients.

Each upsampling block mirrors the corresponding encoder block, with convolutional layers followed by ReLU activations. After the concatenation, a 20% dropout rate is applied to regularize the model and further prevent overfitting. The final layer consists of a 1 × 1 convolution to produce a segmented output map with two classes, followed by a SoftMax activation function, which generates a probability map.

As shown in Table 1, the UNET model was compiled using the Adam optimizer, with a learning rate of 1e^-4^. Categorical cross-entropy was used as the loss function, which is suitable for multi-class segmentation tasks. This configuration ensures the network learns to classify each pixel into the correct class accurately. The model performance was evaluated using standard segmentation metrics, like accuracy and intersection over Union (IoU), which measures the overlap between the predicted and PlanetScope (3m) burnt area masks.

Callback functions were incorporated into the training to optimize the model further. Model Checkpoint was used to restore the best model’s weights based on when the validation intersection over the union score reached the highest point, making sure the best-performing model retained. Another implemented callback was Reduced Learning Rate on Plateau, which monitored the validation Intersection over Union (IOU) score and reduced the learning rate by a factor of 0.9 when the performance of the model did not improve after 10 epochs, with the minimum learning rate being 1e^-7^. Using Reduced Laerning Rate on Plateau helps to get the best out of the model whenever the performance plateaus by tweaking the learning, allowing the model to converge and avoiding overfitting. By incorporating these training strategies, the model achieved stable convergence and avoided overfitting, making it a reliable architecture for semantic segmentation tasks.

### UNET-GRU

**Fig 3:**
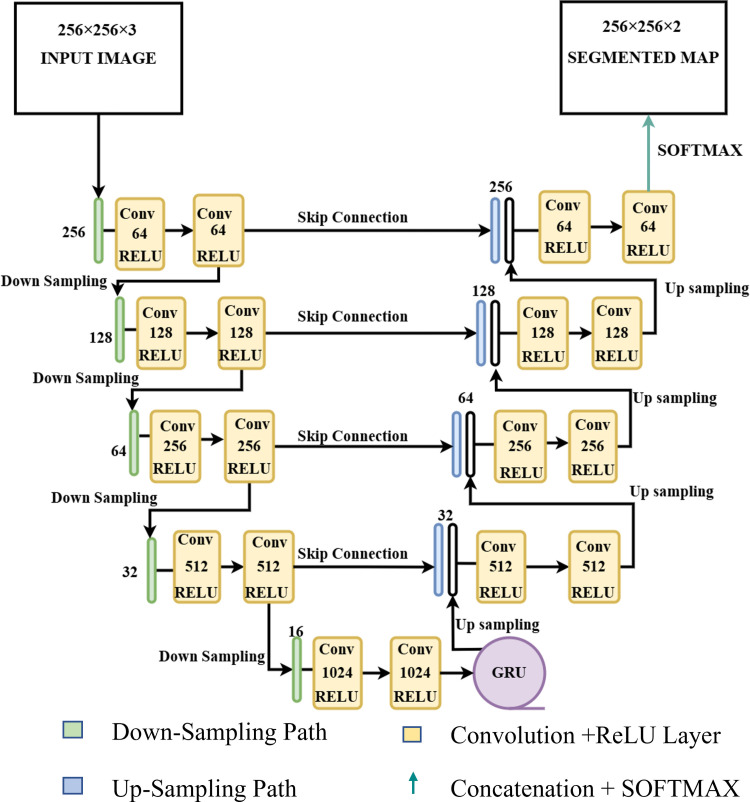
Custom UNET-GRU architecture used in the training process with the encoder, decoder, filters, and activation functions.

We also propose a novel architecture in this study, combining custom UNET with an integrated Gated Recurrent Unit (GRU) (Fig 3). GRU helps the model capture spatial dependencies and contextual information. The UNET-GRU model’s architecture is carefully tailored for pixel-wise classification, like the semantic segmentation tasks in remote sensing; however, the UNET-GRU model is equally capable of delivering precise structure delineation in other domains as well.. The model takes the input with size (256 × 256 × 3) corresponding to height, width, and number of channels.

The encoder is a contracting path where the image downsampling and feature extraction occur. The input layer processes images of size 256 × 256 with three channels. The encoder has four convolutional blocks, where each block has two 3x3 convolutional layers, followed by ReLU activation and He normal weight initialization for adequate gradient flow. Each convolution block is followed by a max-pooling operation with a size of 2 × 2 to reduce the spatial dimensions by a factor of 2. The number of filters after each encoder step doubles, starting from 64 and reaching 512 by the fourth block, ensuring that the model captures critical hierarchical features. A dropout rate of 20% is applied after each convolutional block to prevent overfitting, as shown in (Fig 3). An additional convolutional block with 1024 filters captures the most complex features at the bottleneck.

After the bottleneck, the feature map is reshaped into a 2D tensor. This 2D tensor can be further treated as a sequence that the GRU layer can process. The GRU layer has 512 units and processes the sequence to ensure that the model retains and propagates the spatial information across the feature maps. After processing the GRU layer, the model is reshaped back into the original spatial dimensions and entered by the decoder.

The decoder, or expansive path, mirrors the encoder’s structure which consists of four upsampling blocks, each starting with a 2 × 2 transposed convolutional layer for upsampling and followed by two convolutional layers. Each upsampling step increases the spatial dimensions of the feature maps and reduces the number of filters, starting from 512 and halving until it reaches 64 in the final block.

As shown in (Fig 3), the decoder utilizes skip connections, which concatenate the feature maps from the corresponding encoder blocks to preserve spatial information from the downsampling path. This method helps protect spatial information and improves the gradient flow while maintaining higher segmentation accuracy. A 20% dropout rate is applied after each concatenation operation.

The final output layer is a 1 × 1 convolutional layer followed by a SoftMax activation function. SoftMax converts the feature maps into probability maps, indicating the class for each pixel. The output has a shape of 256 × 256 × 2, representing two classes for segmentation.

In our modified UNET-GRU model, we used the Adam optimizer with a learning rate 1e^-4^. The UNET-GRU is compiled using the categorical cross-entropy loss function, which is ideal for multi-class segmentation tasks. The model performance is evaluated using two key metrics: accuracy and Intersection over Union (IoU), both of which provide insights into the model’s ability to correctly segment the input images, as shown in Table 1. GRU retains the sequential information and the traditional skip connections from the UNET’s architecture, which are helpful in tackling complex segmentation tasks.

Callback functions were incorporated into the training to optimize the model further, which are listed in Table 1. The model checkpoint was used to restore the best model’s weights based on when the validation intersection over the union score reached the highest point. Model Checkpoint retained the best-performing model. Another implemented callback was reducing the learning rate on the plateau, which monitored the validation IOU score and reduced the learning rate by a factor of 0.9 when the model’s performance did not improve even after 10 epochs, with a minimum learning rate of 1e^-7^. Using these callbacks, the model achieves stable convergence and optimal performance as reflected in the evaluation metrics. This approach helped the model overcome common training challenges and maximize its segmentation capabilities.

**Fig 4:**
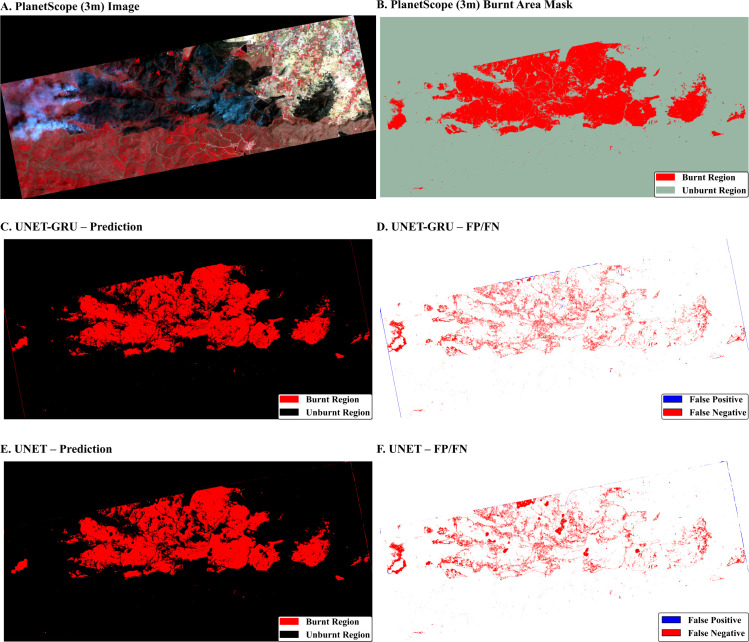
Comparison of burnt area classification using UNET and UNET-GRU models. **(A).** Input PlanetScope satellite imagery; **(B)** Burnt area exclusive mask used for training and validation; **(C)**. Prediction output from UNET-GRU model; **(D)**. Error mask for the UNET-GRU prediction; **(F)**. Prediction output from the UNET model; **(E)**. Error mask for the UNET prediction.

The experiments were conducted on a workstation with 32 GB RAM, an NVIDIA RTX Ada 2000 GPU with 8GB VRAM, using a batch size of 4. The average training time per epoch was approximately 8 seconds for Custom UNET and 21 seconds for UNET-GRU. The GRU enhanced models incurred an additional computational cost due to their complex and deep architecture, as shown in Fig 4; this remained well within the limits. As summarized in Table 1, architecture-specific optimization strategies helped the models achieve efficient convergence. This configuration reflects a balanced trade-off between model complexity, accuracy, and scalability for large-scale remote sensing applications.

### Testing and validation of UNET and UNET-GRU models

The proposed models, Custom UNET and UNET-GRU, were evaluated through comprehensive testing and validation procedures. Both models were evaluated on metrics like Accuracy, Mean Intersection over Union (IoU), F1-Score, Precision, Recall, ROC-AUC, and a Confusion Matrix. These metrics provided a detailed understanding of each model’s performance on the unseen data, while the validation step further reinforced their generalization capabilities across diverse image features.

### Data augmentation and evaluation

A data augmentation technique was used to enhance the robustness of the training process. An image generator was used to create batches of training images along with their corresponding reference masks. The data augmentation technique included parameters like horizontal flips,vertical flips, rotation range of 30 degrees, zoom range of 0.2, fill mode set to reflect, width and height shift range of 0.1 as shown in Table 2. Augmentation introduced spatial and scale variability helping the model generalize better on the unseen data.

**Table 2:** Data augmentation parameters used during training.

Augmentation Type	Parameter
Horizontal Flip	True
Vertical Flip	True
Rotation Range	30 degrees
Width Shift Range	0.1
Height Shift Range	0.1
Zoom Range	0.2
Fill Mode	Reflect

For evaluation, a held-out validation set was used without augmentation. The respective class labels were extracted by processing the predicted and reference masks (PlanetScope (3m) burnt area masks), which were then used to calculate the metrics. This technique helped the models generalize better by introducing randomness to the validation data. The labeled data on burnt and unburnt area patches came from the PlanetScope data (Fig 4B). A Sentinel-2 derived 10-meter (10m) forest mask was applied over the PlanetScope image to ensure only forested regions were considered for burnt area delineation. This forest mask excluded other non-forest classes such as urban surfaces, water bodies, and bare soil.

A new shape file was configured in QGIS with a point and polygon geometries over visually identified burnt and unburnt areas, constrained to the masked forest regions. This shapefile helped to collect numerous precise samples across the image. A meticulous selection process based on manual inspection of spatial patterns and spectral signatures was employed to ensure high accuracy in the selected samples. The PlanetScope (3m) imagery used was in surface reflectance format, and no additional atmospheric correction was applied.

After collecting the samples, the shapefile was processed using the Sample Raster Values feature of QGIS. Sample Raster Values allowed us to analyze the raster values at the selected points and played a crucial role in determining the characteristics of the sampled data. Each band’s minimum and maximum spectral values were noted after this step. A thresholding technique was applied with these values using the Raster Calculator of QGIS, which generated the burnt area mask. The mask was again thoroughly checked through visual interpretation for any discrepancies. This reliable PlanetScope (3m) burnt area mask was used for model training and validation, as shown in (Fig 4B).

## Results

### Burnt area statistics

**Fig 5:**
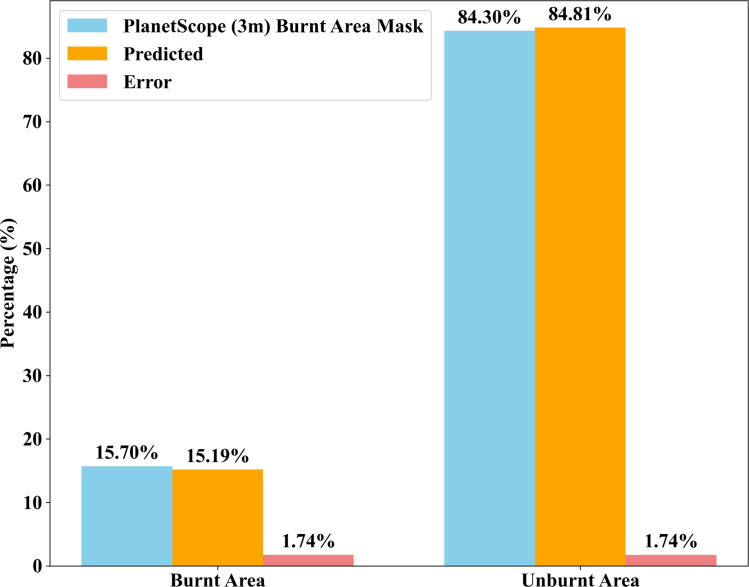
Comparison of burnt and unburnt areas using Custom UNET architecture.

**Fig 6:**
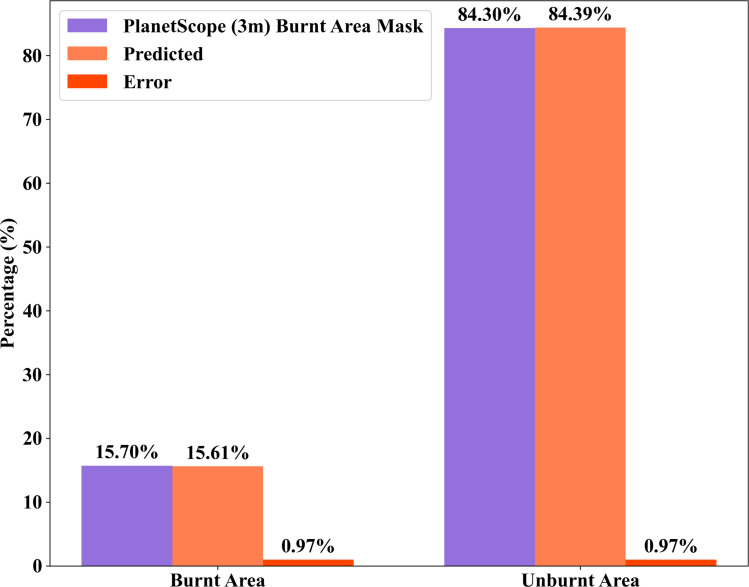
Comparison of burnt and unburnt areas using UNET- GRU architecture.

The comparison between the PlanetScope (3m) burnt area mask and the predictions from UNET and UNET-GRU reveals the precision of both models in classifying burnt and unburnt areas in Tables 3–6, and Figs 5 and 6. Based on the PlanetScope (3m) burnt area mask, 15.7% of the area is burnt, while 84.3% remains unburnt. The UNET model predicted 15.19% as burnt and 84.81% as unburnt, showing a close alignment with this distribution, with only a minor difference of 0.51 percentage points for the burnt category. Similarly, the UNET-GRU model predicted 15.61% as burnt and 84.39% as unburnt, demonstrating an even closer match in the burnt category with a negligible difference of 0.09 percentage points. Both models accurately represent the unburnt areas, with their predictions nearly matching the PlanetScope (3m) burnt area mask proportion of 84.39%. These results highlight the strong performance of both UNET and UNET-GRU, with the UNET-GRU model showing a slightly better accuracy in identifying burnt regions. This marginal improvement in UNET-GRU’s predictions suggests its enhanced capability to handle finer distinctions in burnt area classification.

**Table 3:** Pixel counts and area (in hectares) of burnt and unburnt regions in training, test, and validation sets.

Category	Burnt (Pixels)	Burnt Area (in ha)	Unburnt (Pixels)	Unburnt Area (in ha)
Train	3,711,195	3,340.08	3,170,085	2,853.08
Test	655,000	598.50	491,879	442.69
Validation	655,001	589.50	491,880	442.69

**Table 4:** Confusion matrices for Custom UNET and UNET-GRU for burnt area classification using Bandipur PlanetScope data (values in pixels).

Model	True Negative	False Positive	False Negative	True Positive
UNET	27,101,423	199,264	365,344	4,719,050
UNET-GRU	27,157,940	142,747	172,679	4,911,715

**Table 5:** Normalized Confusion matrices for Custom UNET and UNET-GRU for burnt area classification using Bandipur PlanetScope data (values in pixels) where TN referes to True Negatives, FP refers to False Positives, FN refers to False Negatives, TP referes to True Positives respectively.

Model	TN (Normalized)	FP (Normalized)	FN (Normalized)	TP (Normalized)
UNET	0.837	0.006	0.011	0.146
UNET-GRU	0.839	0.004	0.005	0.152

**Table 6:** Custom UNET and UNET-GRU performance metrics for burnt area classification using Bandipur PlanetScope data.

Metrics	Custom UNET	UNET-GRU
Precision	0.959	0.971
Recall	0.928	0.966
F1-Score	0.943	0.967
Accuracy	0.983	0.990
Mean IoU	0.893	0.940
Dice Coefficient	0.944	0.967

**Fig 7:**
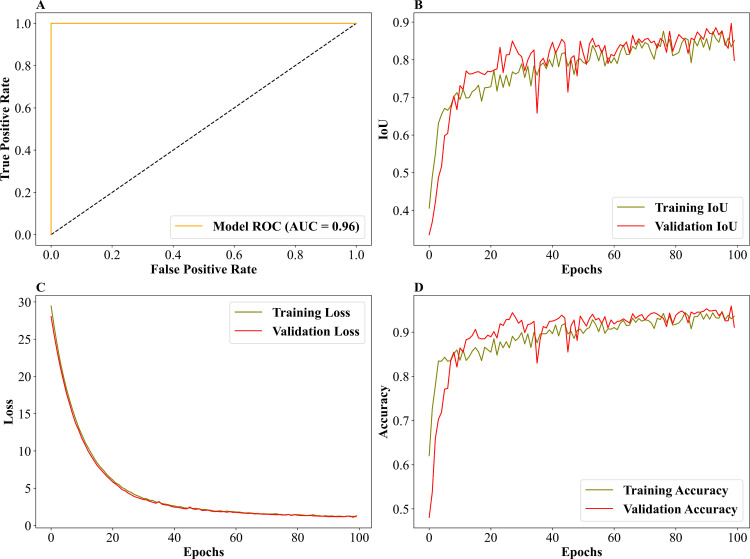
Evaluation metrics for the custom UNET model. (A). Receiver Operating Characteristic–Area Under the Curve (ROC–AUC) for model performance.(B). Intersection over Union (IoU) scores for training and validation datasets across 100 epochs.(C). Training and validation data loss values throughout 100 epochs.(D). Accuracy scores for both training and validation of 100 epochs.

The burnt area classified image from the custom UNET algorithm is given in (Fig 4E), and the performance and behavior metrics are in (Fig 7A–7D). The Receiver Operating Characteristic (ROC) Curve (Fig 7A) plot evaluates the model’s ability to distinguish between classes by plotting the actual positive rate (sensitivity) against the false positive rate (1 − specificity) at various threshold levels. The curve’s area under the ROC (AUC) is 0.96, indicating excellent classification performance. A perfect model would have an AUC of 1.0, while a random classifier would produce a diagonal line (AUC = 0.5). The curve’s closeness to the top-left corner signifies the model’s strong predictive capability, with a low rate of false positives and a high rate of true positives. The training and validation Intersection over Union (Fig 7B) is a spatial metric that measures the overlap between predicted and PlanetScope (3m) burnt areas. Both training and validation IoU improved steadily during the initial epochs and stabilized at high values after approximately 30 epochs. The overlap between the training and validation curves indicates minimal overfitting, as the model generalizes well across unseen validation data. The training and validation loss plot is given in (Fig 7C), showing the loss values for training and validation datasets over 100 epochs. The loss represents the model’s error, and its steady decline indicates effective learning. The close alignment between the training and validation loss curves suggests the absence of significant overfitting, as the model performs similarly on both seen and unseen data. The decreasing trend shows that the model optimizes its predictions as training progresses. Finally, the training and validation accuracy (Fig 7D) plot displays the accuracy achieved by the model on the training and validation datasets over the epochs. Both curves show a sharp increase during the initial epochs, plateauing at high accuracy levels after around 30 epochs. The similarity between training and validation accuracy further supports the conclusion that the model generalizes well and is not overfitted. The final accuracy values, remaining consistently high, reflect the model’s effectiveness in correctly classifying the data. Together, these plots (Fig 7A–7D) present a comprehensive evaluation of the Custom UNET model’s performance, highlighting its strong classification ability (high AUC), spatial prediction accuracy (IoU), error minimization (loss), and reliability in generalization (accuracy).

**Fig 8:**
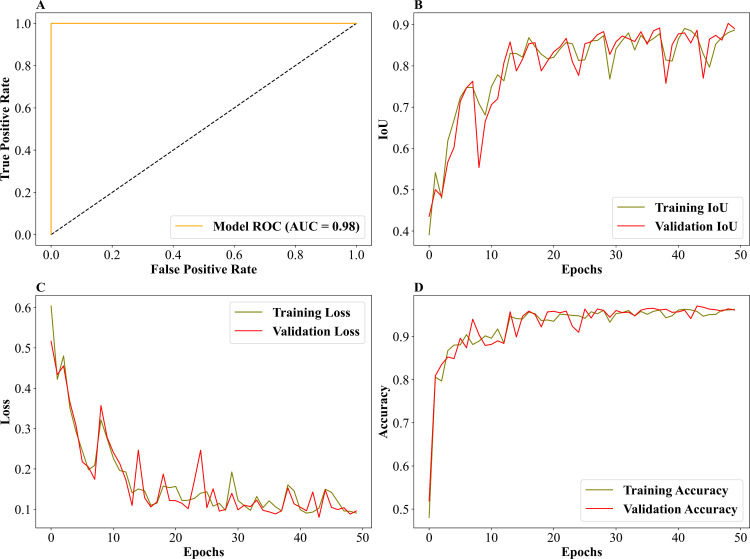
Evaluation metrics for the custom UNET-GRU model. (A). Receiver Operating Characteristic–Area Under the Curve (ROC–AUC) for model performance.(B). Intersection over Union (IoU) scores for training and validation datasets over 50 epochs.(C). Loss values for training and validation data across 50 epochs.(D). Accuracy scores for both training and validation over 50 epochs.

The burnt area classified image from the custom UNET-GRU algorithm is given in (Fig 4C), and the performance and behavior metrics provide the performance during model training and evaluation. In (Fig 8A), the ROC area under the curve is 0.98, which is highly indicative of excellent classification performance. The curve is very close to the top-left corner, signifying that the UNET-GRU model effectively minimizes false positives while maximizing true positives, outperforming random or mediocre classifiers (AUC = 0.5). The training and validation loss plot (Fig 8C) tracks the loss values for training and validation datasets over 50 epochs. The steady decrease in loss indicates that the model successfully minimizes error as training progresses. The training and validation loss curves remain closely aligned throughout, highlighting that the model generalizes well and avoids significant overfitting. By the end of training, both losses stabilize at low values, reflecting the model’s ability to make accurate predictions. (Fig 8B) on training and validation indicates that both the training and validation IoU values steadily increase during the initial epochs and stabilize above 0.9, showcasing the model’s strong spatial prediction capabilities. The minimal gap between the training and validation IoU curves emphasizes the model’s robustness and consistency in handling unseen validation data. Finally, the training and validation Accuracy (Fig 8D) represents the model’s accuracy for training and validation datasets over the epochs. Both curves exhibit rapid improvement during the initial epochs, reaching and stabilizing at high accuracy levels (above 0.9) early in the training process. The close alignment between the two curves demonstrates that the model achieves consistently high accuracy on both seen and unseen data, further supporting its generalizability. Overall, the plots (Fig 8A–8D) collectively highlight the superior performance of the UNET-GRU model, with a high AUC, low loss, high IoU, and substantial accuracy. The minimal divergence between training and validation metrics underscores the model’s effective learning process and ability to generalize across datasets.

### Comparative model performance metrics

The confusion matrix offers further insights into the model’s classification performance, indicating how pixels from the predicted masks were classified into their respective classes. The confusion matrices and normalized confusion matrices shown in Tables 4 and 5 for both UNET and UNET-GRU models refer to the number of true positives, false positives, true negatives, and false negative, crucial for calculating Precision, Recall, Accuracy, and other performance metrics as referenced in Table 6. Precision, a measure of the proportion of correctly predicted positive observations to total predicted positives, highlights the advantage of UNET-GRU, as its higher value indicates a better ability to minimize false positives compared to the Custom UNET. While both models perform well in this aspect, UNET-GRU demonstrates slightly greater precision. Recall, which evaluates the proportion of correctly predicted positive observations to all actual positives, showed a significant edge for UNET-GRU, as it more effectively captured true positives and minimized false negatives. The F1-Score, representing the harmonic mean of Precision and Recall, consolidates these strengths; the UNET-GRU’s higher F1-Score reflects its superior overall performance in scenarios where false positives and false negatives are critical. Similarly, accuracy, which measures the proportion of correctly predicted observations (both positives and negatives) to total observations, reveals that UNET-GRU makes fewer total prediction errors than the Custom UNET, although by a small margin. For spatial metrics, the Mean IoU (Intersection over Union), a measure of overlap between predicted and PlanetScope (3m) burnt area mask, demonstrates a significant improvement for UNET-GRU, suggesting that its predictions align more closely with the labeled images, ensuring better spatial accuracy. The Dice Coefficient, another overlap measure sensitive to small regions, mirrors this trend, further confirming UNET-GRU’s ability to predict burnt areas accurately.

Across all metrics, i.e., Precision, Recall, F1-Score, Accuracy, Mean IoU, and Dice Coefficient, the UNET-GRU outperformed the Custom UNET, with notable improvements in Recall (3.8% higher), Mean IoU (4.66% higher), and F1-Score (2.53% higher). These differences in metrics like Recall, Mean IoU, F1-Score highlight UNET-GRU’s superior capability in identifying positive cases and achieving spatial consistency with the labeled data. While the Custom UNET performed strongly as an effective baseline, integrating GRU with UNET, as UNET-GRU, likely enhanced its ability to capture sequential or contextual information, improved performance in tasks that benefit from temporal or spatial dependencies. Our UNET-GRU emerges as the more robust and preferred choice for applications requiring precise and reliable predictions.

### Qualitative assessment of predictions

**Fig 9:**
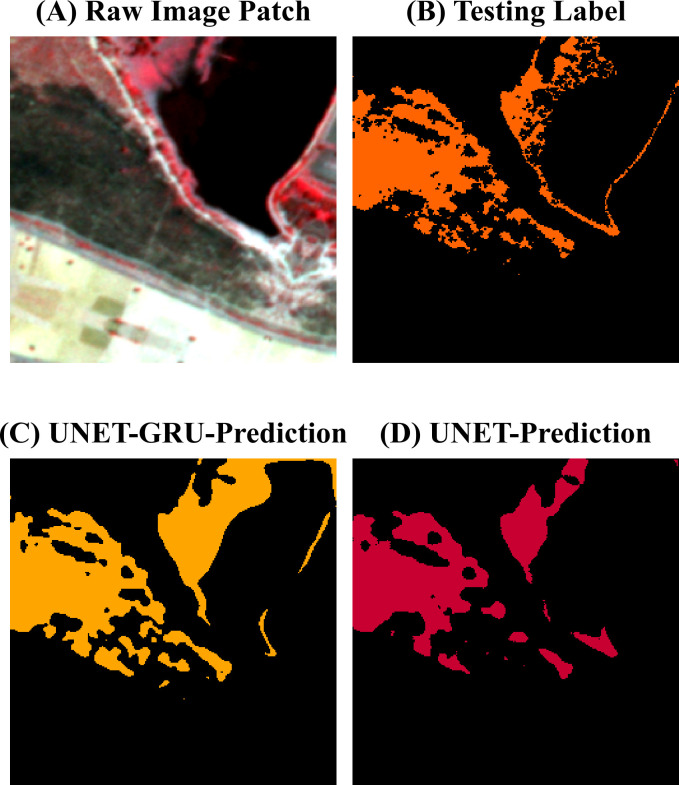
Evaluation of burnt area classification performance near a water body. (A). Raw image patch showing a water body surrounded by vegetation and burnt areas. (B). PlanetScope (3m) burnt area mask indicating burnt areas in orange and unburnt areas in black. **(C)**. The prediction from the UNET-GRU model captures most burnt regions but misclassifies parts of the water body as burnt. (D). The custom UNET model’s prediction avoids water body misclassification but underestimates finer burnt patches.

**Fig 10:**
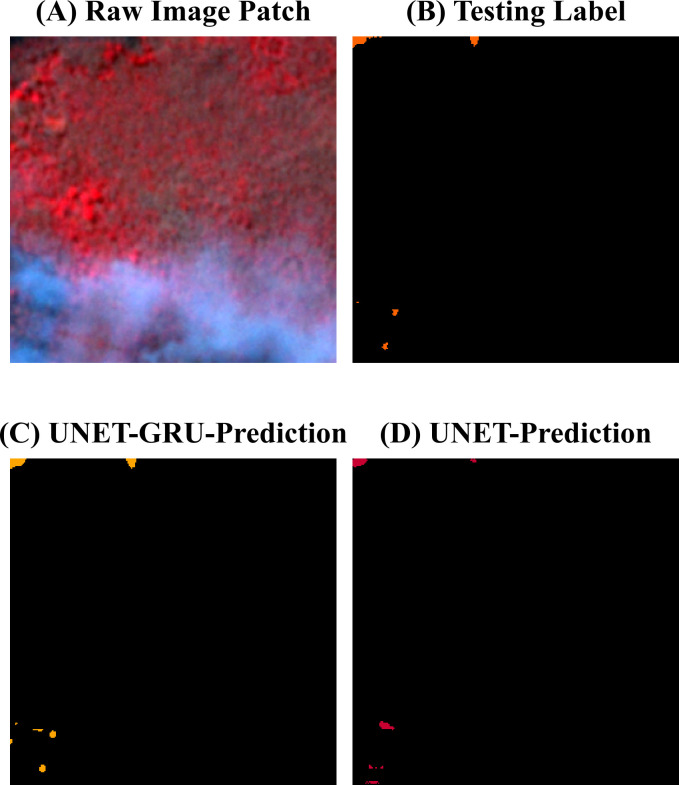
Evaluating Burnt area classification on sparse burnt regions with minimal PlanetScope (3m) burnt area labels. (A). Raw image patch showing a sparsely burnt area with minimal PlanetScope (3m) burnt regions.(B). Testing label indicating the PlanetScope (3m) for burnt areas in orange, with very few burnt regions.(C). Prediction from the UNET-GRU model, which identifies some of the small burnt areas but introduces false positives.(D). Prediction from the Custom UNET model, which misses most of the small burnt areas, showing higher specificity but lower sensitivity.

**Fig 11:**
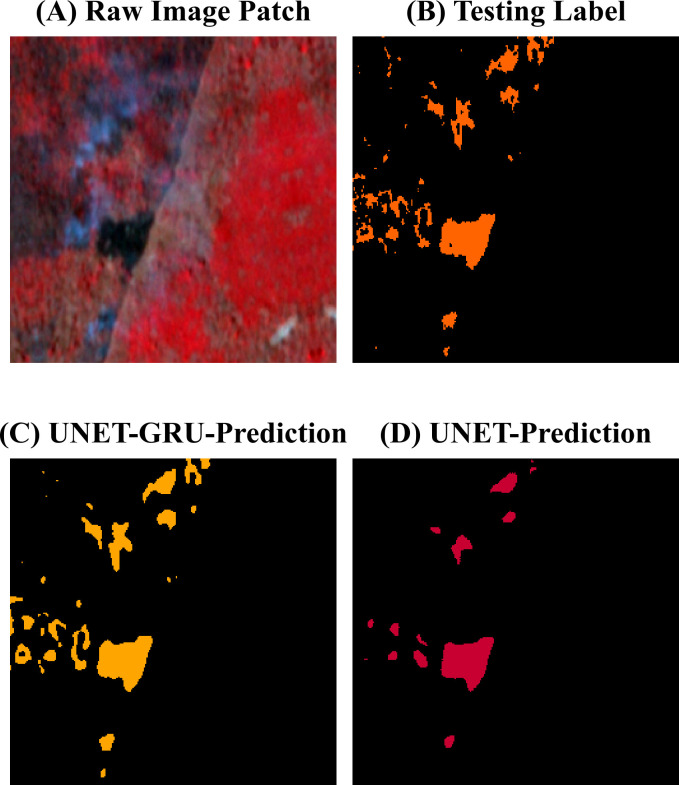
Evaluating Burnt area classification on fragmented burnt regions with smaller patches. (A). Raw image patch showing a region with clear burnt areas amidst vegetation.(B). Testing label indicating the PlanetScope (3m) burnt regions in orange, highlighting the accurate burnt areas.(C). Prediction from the UNET-GRU model, which successfully identifies most of the burnt regions, but over-predicts some boundaries, leading to slight noise.(D). Prediction from the Custom UNET model, which is more conservative, successfully capturing the burnt areas but underestimating the extent, particularly in fragmented burnt regions.

Visual comparisons between model predictions offer further insight into the segmentation behavior of the UNET-GRU and Custom UNET models. (Fig 9) shows forecasts for a region containing a water body adjacent to vegetation and burnt areas. The UNET-GRU model accurately captures most burnt regions but also misclassifies parts of the water body as burnt. In contrast, the Custom UNET avoids this misclassification but fails to detect finer, fragmented burnt patches. Fig 10 presents a sparsely burnt area with minimal PlanetScope (3m) burnt area labels. The UNET-GRU detects several small burnt regions but introduces false positives, while the Custom UNET, though more specific, misses the majority of these patches. In (Fig 11), which depicts a scene with small, disconnected burnt patches, UNET-GRU again captures the burnt areas more comprehensively but tends to over-fit and include noise. The Custom UNET misses most of the fine-scale burnt areas, underestimating the extent of burnt regions. These examples highlight the models’ contrasting behaviors, particularly in challenging scenarios involving spectral confusion or sparse labels. A detailed analysis of these patterns is provided in the Discussion section.

### Statistical significance of ROC-AUC improvements

To determine whether the performance differences between the Custom UNET and UNET-GRU models were statistically significant, tile-wise ROC-AUC scores were calculated across the validation set with 35 image-mask pairs. The mean ROC-AUC of the Custom UNET model was 0.9794, while UNET-GRU attained a higher mean of 0.9943.

**Fig 12:**
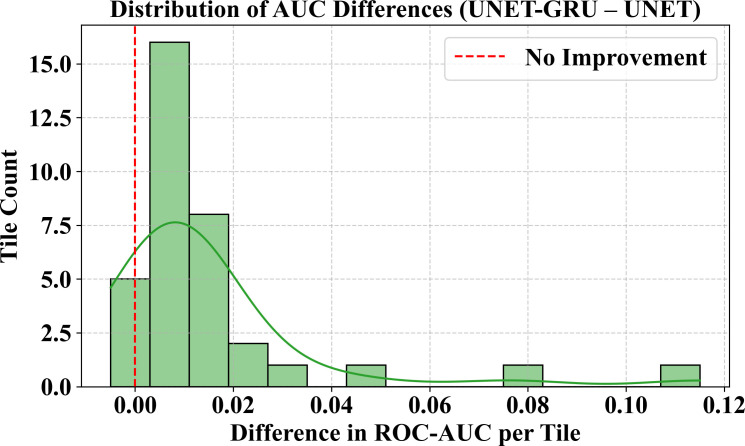
Distribution of AUC differences between UNET-GRU and UNET models. Histogram showing the tile-wise differences in ROC-AUC scores between the UNET-GRU and Custom UNET models. Most of tiles exhibit a positive AUC difference, indicating consistent improvement by the UNET-GRU model across the validation dataset. A red dashed line at zero marks the point of no improvement. The histogram is overlaid with a kernel density estimate (KDE) to highlight the skewness of the distribution, favoring UNET-GRU.

**Fig 13:**
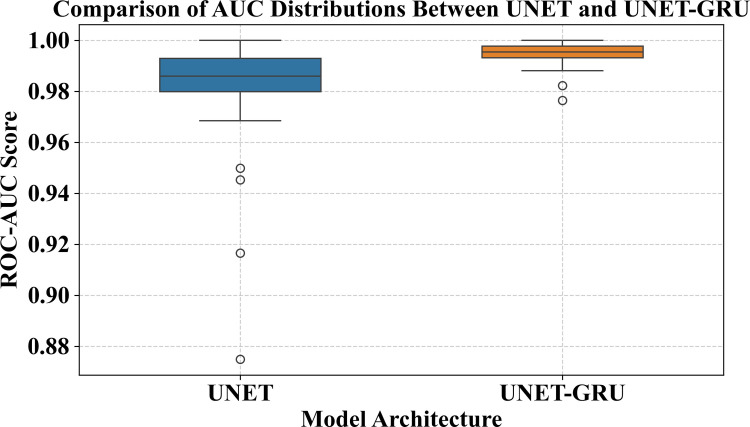
Boxplot comparison of AUC scores for UNET and UNET-GRU models. Boxplot showing the distribution of tile-wise ROC-AUC scores for the Custom UNET and UNET-GRU models. The UNET-GRU model achieves a higher median AUC and exhibits reduced variability across validation tiles, with fewer outliers than the baseline UNET. This suggests improved consistency and generalization in the segmentation of burnt areas.

A paired t-test was conducted to assess whether the mean difference in AUC scores between the two models differed significantly from zero. The paired t-test produced a t-statistic of 3.85 and a corresponding p-value of 0.000494, which indicates a statistically significant difference at the 1% level, assuming normality in the differences. A Wilcoxon signed-rank test was also performed to further validate this result without relying on parametric assumptions. The Wilcoxon test got a test statistic of W = 24.0 with p-value of 4.44 × 10 ⁻ ⁸, which confirmed that the observed performance improvement is statistically significant.

(Fig 12) shows the distribution of per-tile AUC differences. The majority of values to the right of zero demonstrate that the UNET-GRU model provides continuous performance gains. Furthermore, (Fig 13) shows a boxplot comparison of AUC ratings for both models. UNET-GRU indicates a higher median AUC, fewer outliers, and lower variability, indicating increased robustness and stability over spatially diverse regions.

Together, these statistical tests and visual analyses confirm that the UNET-GRU model consistently and significantly outperforms the Custom UNET in classifying burnt areas. The enhanced performance and reduced variability of the UNET-GRU across spatially diverse tiles emphasize its reliability and effectiveness, reinforcing its potential for operational deployment in high-resolution remote sensing applications.

## Discussion

The comparative analysis between Custom UNET and UNET-GRU models for burnt area classification highlights the strengths and limitations inherent to each approach. Both models demonstrated high accuracy in distinguishing burnt and unburnt areas, with UNET-GRU achieving superior results across most performance metrics, including Accuracy, Precision, Recall, F1-Score, Mean IoU, and Dice Coefficient. The ROC curves underscore this, with UNET-GRU achieving an AUC of 0.98 compared to 0.96 for Custom UNET, showcasing its enhanced ability to minimize false positives while maintaining high classification performance.

Comparing the results from this study with previous studies reveals notable similarities and advancements. Kim et al. [53] reported F1-scores of 0.964–0.965 and IoUs of 0.938–0.942 using a UNET model on PlanetScope imagery, while [54] achieved F1-scores exceeding 0.90 for unitemporal PlanetScope imagery. In contrast, our UNET-GRU model achieved a Mean IoU of 0.95 and an F1-score of 0.975, indicating its superior ability to model spatial and contextual dependencies. The lower training and validation losses observed during the training process further support its robustness and generalizability.

The enhanced performance of UNET-GRU aligns with the findings of Gonçalves et al. [55], who emphasized the importance of capturing contextual relationships in burned area mapping. While Transformers have shown promise in this domain, the GRU integration within our UNET architecture offers comparable benefits in contextual modeling, as evidenced by the high Recall and Dice Coefficient scores in this study.

In addition to transformer-based methods, recent approaches have explored a hybrid CNN-attention architecture, such as Swin-Unet [56] and SwarmNet [57], which have demonstrated strong performance on high-resolution imagery but are computationally expensive. Vision Transformer (ViT)–based models incorporating spectral–spatial attention mechanisms [58] have further improved feature discrimination. However, they tend to be sensitive to the volume and variability of training data while also being computationally expensive.

Siamese and change detection networks have become increasingly popular for mapping burnt areas, particularly when pre- and post-fire images are available. Wang et al. [59] proposed a dual-stream Siamese model that integrates optical and SAR data, demonstrating high accuracy but requiring precisely aligned multi-temporal inputs. Similarly, Li et al. [60] employed NDVI differencing in combination with deep Siamese networks to detect temporal changes. While these models are effective, their reliance on multi-temporal imagery can limit their use in rapid-response scenarios. In contrast, our UNET-GRU model achieves state-of-the-art performance using only a single post-fire image, offering greater flexibility and operational scalability for near-real-time burnt area mapping.

Comparative insights between the studies are summarized in Table 7, which presents a side-by-side overview of model architectures, input types, and key performance metrics across different studies. Among all compared methods, our UNET-GRU model achieved the highest F1-score and Mean IoU using only a single post-fire PlanetScope image, demonstrating both state-of-the-art segmentation performance and operational efficiency, which can be attributed to the GRU’s ability to directly capture contextual and spatial dependencies from raw spectral reflectance data. However, relying solely on spectral cues from the available bands can introduce limitations in cases of spectral ambiguity, such as misclassifying water bodies as burnt areas. This misclassification suggests the potential value of incorporating additional features like spectral indices or auxiliary data sources in future work.

**Table 7:** Comparative performance of state-of-the-art models for burnt area mapping.

Study	Model	Input Type	Mean IoU	F1-Score
Wang et al. [59]	Siamese Dual-Stream	Optical + SAR	0.91	0.94
Cho et al. [54]	CNN	PlanetScope	0.90	0.90
Gonçalves et al. [55]	Transformer	PlanetScope (post-fire)	0.94	0.96
Zhou et al. [57]	SwarmNet	Sentinel-2	0.92-0.94	0.95
Xie et al. [58]	ViT + Attention	Sentinel-2	0.93	0.94
Kim et al. [53]	UNET	PlanetScope (post-fire)	0.938-0.942	0.964-0.965
Current Study	UNET-GRU	PlanetScope	0.95	0.975

Despite the overall superior performance, certain limitations of UNET-GRU must be acknowledged. The UNET-GRU model exhibited occasional false positives, particularly when distinguishing spectrally similar features like water bodies and burnt regions, as observed in (Fig 9C) where portions of a water body were misclassified as burnt areas. This misclassification likely results from overlapping spectral characteristics and insufficient training data to refine boundary differentiation. Furthermore, while UNET-GRU demonstrated high sensitivity to burnt regions, it also overestimated boundaries in some instances, leading to noisy predictions, as evident in (Fig 9C), where smaller fragmented burnt patches were over-predicted.

In contrast, the Custom UNET model showed higher precision and was better at avoiding false positives, especially around water and cloud-shadow regions (Fig 9D). However, higher precision had its trade off as it missed smaller burnt features, as seen in Fig 10D. The Custom UNET model failed to detect many fine-scale patches that were present in the PlanetScope (3m) burnt area mask, causing an underestimation of the burnt area, suggesting a limitation in its ability to generalize to less prominent patterns. Both models also faced challenges in preserving the boundary details of burnt regions, with the UNET-GRU occasionally producing blurred or expanded edges and the Custom UNET producing overly smooth segmentations.

The results from this study highlight a trade-off between the sensitivity and specificity of the models. The UNET-GRU model demonstrated strong sensitivity to burnt areas by successfully capturing both large and fragmented patches Figs 9C and 11C. However, the UNET-GRU model also produced false positives in regions with spectrally similar features like water bodies, which were misclassified as burnt areas. The misclassification of pixels is particularly evident in (Fig 9C) which is likely due to the spectral overlap in the NIR, Red bands and limited representation of such examples in the training data. Additionally, the UNET-GRU model often over-segmented smaller burnt patches, leading to fragmented and less precise boundaries, and in some cases, an overestimation of the affected area.

On the other hand, the Custom UNET model showed higher precision and was better at avoiding false positives, especially around water and cloud-shadow regions (Fig 9D). However, higher precision had its trade off as it missed smaller burnt features, as seen in (Fig 10D). The Custom UNET model failed to detect many fine-scale patches that were present in the PlanetScope (3m) burnt area mask, causing an underestimation of the burnt area, suggesting a limitation in its ability to generalize to less prominent patterns. Both models also faced challenges in preserving the boundary details of burnt regions, with the UNET-GRU occasionally producing blurred or expanded edges and the Custom UNET producing overly smooth segmentations.

To address the identified limitations, future work will incorporate additional spectral indices, such as NDVI and NDWI, to improve the models’ ability to distinguish between spectrally similar classes. Increasing the diversity of the training dataset by incorporating more edge cases such as water-proximate fires and fragmented burn patterns could improve the model’s overall robustness. In addition to this, post-processing techniques like Conditional Random Fields [61] or edge-aware refinement methods [62,63], will be explored to improve segmentation quality along burnt region boundaries. Finally, combining the strengths of both UNET-GRU and Custom UNET models through ensemble approaches may provide a more balanced solution, leveraging the sensitivity of UNET-GRU and the specificity of Custom UNET. These steps align with findings from recent studies addressing similar challenges in burned area mapping [51–54].

## Conclusions

The comparative analysis of the Custom UNET and UNET-GRU models highlights their strong performance in burnt area classification, with both models demonstrating close alignment to the labeled PlanetScope original data. While the Custom UNET serves as a robust baseline, integrating GRU into the UNET architecture (UNET-GRU) resulted in notable improvements across key metrics, including Recall, F1-Score, Mean IoU, and Dice Coefficient. The improvements across various metrics highlight the UNET-GRU’s ability to capture subtle distinctions and handle spatial dependencies, leading to more accurate and consistent predictions.

The high AUC values (0.96 for UNET and 0.98 for UNET-GRU) further validate the strong classification capabilities of both models, with UNET-GRU achieving a slight edge in minimizing false positives and maximizing true positives. The performance difference between UNET and UNET-GRU was found to be statistically significant, as confirmed by both a paired t-test (*p* = 0.000494) and a Wilcoxon signed-rank test (*p* = 4.44 × 10 ⁻ ⁸), further showing the robustness and generalizability of UNET-GRU across spatially diverse validation tiles.

The minimal divergence between training and validation metrics observed in both models confirms their effective learning processes and strong generalization to unseen data. For applications requiring precise spatial predictions and reliability, the UNET-GRU emerges as the more robust and reliable choice, particularly in scenarios that benefit from temporal or contextual information.

These results, supported by high classification metrics like Recall, Accuracy, Precision, ROC-AUC and statistically significant differences between models, provide a strong foundation for advancing research into the performance of these models across a wider variety of datasets and more complex classification tasks. Expanding the scope of investigation to include diverse datasets, such as those from different geographic regions, varying resolutions, and distinct environmental conditions, will enable a deeper understanding of the models’ adaptability and robustness. These efforts are underway to evaluate the generalizability of the UNET and UNET-GRU architectures and to refine their application for broader scenarios.

## Supporting information

S1 File Epoch-level training and validation metrics for the UNET model. This file includes intersection-over-union (IoU), accuracy, and loss values for each training and validation epoch, providing insight into the UNET model convergence behavior.
(CSV)

S2 File Epoch-level training and validation metrics for the UNET-GRU model. This file includes intersection-over-union (IoU), accuracy, and loss values for each training and validation epoch, providing insight into the UNET-GRU model convergence behavior.
(CSV)

S3 File Summary of evaluation metrics for both UNET and UNET-GRU models. This file includes precision, recall, F1-score, accuracy, mean IoU, and Dice coefficient. These metrics support the main quantitative results table presented in the manuscript.
(CSV)

S4 File Tile-wise ROC–AUC scores for the UNET and UNET-GRU models. Each row shows the AUC score for a specific tile in the study area. These values help compare how well each model performed in different locations.
(CSV)

S5 File Confusion matrix of pixel-wise classification results for the UNET and UNET-GRU models. The file includes counts of true positives, false positives, false negatives, and true negatives, which were used to calculate model performance metrics.
(CSV)
